# Analysis of the utility of a rapid vesicle isolation method for clinical strains of *Pseudomonas aeruginosa*

**DOI:** 10.1128/spectrum.00649-24

**Published:** 2024-09-09

**Authors:** Tania Henriquez, Francesco Santoro, Donata Medaglini, Lucia Pallecchi, Ilaria Clemente, Claudia Bonechi, Agnese Magnani, Eugenio Paccagnini, Mariangela Gentile, Pietro Lupetti, Massimiliano Marvasi, Alessandro Pini, Luisa Bracci, Chiara Falciani

**Affiliations:** 1 Department of Medical Biotechnologies, University of Siena, Siena, Italy; 2 Department of Biotechnology, Chemistry and Pharmacy, University of Siena, Siena, Italy; 3 Department of Life Sciences, University of Siena, Siena, Italy; 4 Department of Biology, University of Florence, Florence, Italy; University of Florida, Gainesville, Florida, USA

**Keywords:** extracellular vesicles, *Pseudomonas aeruginosa*, bacterial phenotype, vesicle purification

## Abstract

*Pseudomonas aeruginosa*, a pathogen capable of causing diseases ranging from mild to life-threatening, has a large arsenal of virulence factors. Notably, extracellular vesicles have emerged as significant players in the pathogenesis of this organism. However, the full range of their functions is still being studied, and difficulties related to vesicle purification (long protocols, low yields, and specialized instruments) have become a major obstacle for their characterization. In this context, the utility of rapid new methods of vesicle isolation from clinical strains is still unknown. Here, we analyze the utility of the ExoBacteria OMV isolation kit for a collection of clinical strains of *P. aeruginosa*. We first phenotypically characterized 15 *P*. *aeruginosa* strains to ensure that our samples were heterogeneous. We then determined the best conditions for purifying vesicles from *P. aeruginosa* PAO1 reference strain by the rapid method and used them to isolate vesicles from clinical strains. Our results indicated that M9 minimal medium is the best for obtaining high purity with the rapid isolation kit. Although we were able to isolate vesicles from at least four strains, the low yield and the large number of strains with unpurifiable vesicles showed that the kit was not practical or convenient for clinical strains. Our findings suggest that although fast procedures for vesicle purification can be of great utility for *Escherichia coli*, the more complex phenotypes of clinical isolates of *P. aeruginosa* are a challenge for these protocols and new alternatives/optimizations need to be developed.

**IMPORTANCE:**

*Pseudomonas aeruginosa* is recognized as an opportunistic pathogen in humans and animals. It can effectively colonize various environments thanks to a large set of virulence factors that include extracellular vesicles. Different methods were recently developed to reduce the time and effort associated with vesicle purification. However, the utility of rapid vesicle isolation methods for clinical strains of *P. aeruginosa* (which are recognized as being highly diverse) is not yet known. In this context, we analyzed the utility of the ExoBacteria OMV Isolation kit for vesicle purification in *P. aeruginosa* clinical strains. Our findings showed that the kit does not seem to be convenient for research on clinical strains due to low vesicle recovery. Our results underscore the importance of developing new rapid vesicle purification protocols/techniques for specific clinical phenotypes.

## INTRODUCTION

*Pseudomonas aeruginosa* is an opportunistic pathogen of humans that can survive in a wide range of environments due to its large genome and ample set of virulence factors (1–4). It is best known for infecting immunocompromised people, patients with chronic lung diseases (e.g., cystic fibrosis, non-cystic-fibrosis bronchiectasis, obstructive chronic pulmonary disease), and burn patients (1–5). Understanding the mechanisms by which *P. aeruginosa* establishes infections is imperative for developing effective therapeutic strategies. In this context, the vesicles produced by *P. aeruginosa* and their role in virulence have attracted significant research in the last 10 years (6, 7). Also known as extracellular vesicles (EVs), these vesicles are recognized players in intercellular communication, and bacterial pathogenicity and survival strategies, among others (6). Since vesicles can carry a wide range of biomolecules, such as proteins, lipids, nucleic acids, and virulence factors (8), they function as a highly developed means of delivering bacterial cargos to host cells. Hence, EVs aid immune evasion, modify host responses, and promote the onset and spread of infections (9). Their study is crucial for understanding the range of pathogenicity of *P. aeruginosa* strains.

Extracellular vesicles have also shown promise as carriers for drug delivery, vaccines, and diagnostic tools (6, 10). Unraveling the intricate biology of *P. aeruginosa* vesicles is, therefore, not only crucial for understanding bacterial pathogenesis but is also a key for developing innovative approaches for therapeutic intervention. Ongoing research is focused on elucidating the biogenesis (11), composition (12), and functions (13) of bacterial vesicles, for which vesicle purification is crucial. Unfortunately, the purification of EVs from different samples currently has low yields and is time-consuming, laborious, and expensive. This limits the possibilities of EV research by a wider community and is one reason why, despite their importance, EVs have not been used in research on a larger scale. Various methods are available for the isolation of vesicles; they include differential centrifugation (low-speed and ultracentrifugation), size-exclusion chromatography (SEC), hydrostatic filtration dialysis (HFD), and affinity purification (8). All require specific equipment and/or are time-consuming.

To overcome this obstacle, some fast methods (including commercial kits) have recently been developed. These are simpler and improve purity, reducing the time needed for the procedure. However, the utility of these rapid methods with clinical strains of *P. aeruginosa*, known for their phenotypic variability (14), has not yet been investigated. Here, we studied the utility of the ExoBacteria OMV Isolation Kit (System Biosciences), a rapid method for the purification of vesicles, applied to a heterogeneous collection of clinical strains *of P. aeruginosa*.

## MATERIALS AND METHODS

### Bacterial strains and culture media

*Pseudomonas aeruginosa* strains were grown in King’s B (KB) medium and maintained on cetrimide agar plates. For long-term storage, they were kept in glycerol at −80°C. A complete list of the strains used in this study can be found in Table 1. For growth curves, KB medium [prepared as previously described (15)] and 2xTY [10 g yeast extract, 5 g NaCl, and 16 g peptone per liter, as described by Sambrook and Russel (16)] were used. M9 minimal medium containing 1× M9-salts, 18.7 mM NH_4_Cl, 0.2 mM CaCl_2_, 2 mM MgSO_4_, and 0.4% glucose was prepared. All strains were cultured at 37°C. King A medium (also known as “Pseudomonas Agar P,” #60788, Millipore) and Cetrimide agar (#70887, Millipore) were prepared according to the manufacturer’s instructions.

**TABLE 1:** List of strains used in this study

Name (strains)	Year of isolation	Origin	Source
PAO1	1954	Wound	(17)
ATCC 27853	1971	Blood	(18)
LS01	2023	Bronchoalveolar lavage	This work
LS03	2023	Sputum	This work
LS04	2023	Bronchial aspirate	This work
LS05	2023	Bronchial aspirate	This work
LS06	2023	Urine	This work
LS07	2023	Cerebrospinal fluid	This work
LS08	2023	Tracheal aspirate	This work
LS09	2023	Urine	This work
Z33	2005	Sputum (cystic fibrosis patient)	(19)
Z34	2006	Sputum (cystic fibrosis patient)	(19)
Z37	2008	Sputum (cystic fibrosis patient)	(19)
M1	2002	Sputum (cystic fibrosis patient)	(19)
M25	2002	Sputum (cystic fibrosis patient)	(19)

### Growth experiments

Strains were grown overnight in KB medium with continuous shaking and then used to inoculate a 96-well plate containing 100 µL KB or 2xTY media. The 96-well plate was incubated in a Tecan reader (Infinite 200Pro) for 24 h at 37°C under continuous shaking. OD 600 and pyoverdine fluorescence (excitation 400 nm; emission 455 nm) were read every 30 min. The growth of each strain was analyzed in triplicate. Pyoverdine production was expressed as the highest measurement of relative fluorescence units (RFU) normalized to growth at that specific time point (OD600).

### Vesicle isolation

The fast purification method, ExoBacteria OMV Isolation Kit (System Biosciences), was used. Strains were maintained on cetrimide agar plates and used to inoculate 2 mL of KB medium. The cultures were grown during the day at 37°C under continuous shaking (150 rpm) and used to inoculate 25 mL of M9 minimal medium (incubation at 37°C under shaking). The overnight cultures were used for vesicle isolation with the ExoBacteria OMV Isolation Kit (System Biosciences) according to the manufacturer’s instructions with small modifications. Briefly, cultures were centrifuged twice at 4,000 *g* for 25 min at 4°C, and supernatants were filtered with a 0.22 µm filter. The kit was then used according to the instructions, and the vesicles were recovered in 1 mL of elution buffer.

For ultrafiltration/ultracentrifugation, strains were grown overnight in KB medium and used to inoculate 100 mL of fresh KB or M9 minimal medium. The cultures were then incubated at 37°C under shaking for 4–5.5 h (until they reached exponential phase) and centrifuged for 30 min at 4,000 *g* and 4°C. Later, the protocol described by Tan and colleagues was used with small modifications (20). First, the supernatants were filtered twice with a 0.22-µm filter, followed by processing with an ultrafiltration device (Vivaspin 20, 100 kDa MWCO PES, Cytiva), including buffer exchange to 1× PBS. The samples collected were then stored at −80°C. Finally, the samples were thawed and ultracentrifuged for 1 h at 4°C and 100,000 *g*. The supernatant was discarded, and the pellet was resuspended in 1 mL PBS and stored at −80°C.

### Phenotypic characterization of strains

For pigment production (including pyoverdine) and mucoid phenotype detection, strains were streaked into cetrimide, King A and King B agar plates, and incubated overnight at 37°C. For colony morphology assay, strains were grown in 2xTY, and then, 5 µL was inoculated onto cetrimide and 2xTY agar. Later, the plates were incubated at 37°C, and pictures were taken under visible and UV light. Pyoverdine was also measured during the growth experiments, as previously mentioned. The experiments were performed in triplicate. To analyze hemolytic phenotype, strains were inoculated into TSA with 5% Sheep Blood (Biomerieux) and incubated at 37°C up to 48 h.

### Immunodetection of OprF

Dot blot experiments: Samples directly eluted from the column were spotted onto a nitrocellulose membrane and left to dry for 1 h. The membrane was then blocked (3% BSA in TBS) and incubated on a rocker shaker for 1 h at RT. After that, primary antibody solution (containing anti-OprF, Thermo Fisher PA5-117553, diluted 1:2,500 in TBS +0.05% Tween, 3% BSA) was added and incubated 1 h at RT on a rocker shaker. Later, the solution was removed, and the membrane was washed three times with TBS +0.05% Tween (10 min each time) on a rocker shaker at RT. A secondary antibody solution containing goat anti-rabbit IgG H&L (HRP) (Abcam 97051) diluted 1:20,000 in TBS +5% skimmed milk was added and incubated for 1 h at RT on a rocker shaker. Again, the membrane was washed three times with TBS +0.05% Tween (10 min each time) on a rocker shaker at RT. Finally, the buffer was discarded, and ECL Select Western Blot Detection Reagent kit (Cytiva RPN2235) was added on top of the membrane (according to the manufacturer’s instructions) and incubated for 5 min at room temperature. The final image was captured using a LAS Imager (settings: precision, auto, high resolution, chemiluminescence).

SDS-PAGE and western blot experiments: Vesicle samples washed with 1× PBS and concentrated (~5×) using an Amicon Ultra 0.5 centrifugal filter (100 kDa cutoff) were used for SDS-PAGE (12% mPAGE precast gels (Merck) in 1× MES SDS, 120 V for 50 min) and western blot analysis (following the protocol described for Dot blot). For transfer, a Trans-Blot Turbo Mini 0.2 µm Nitrocellulose Transfer Pack (Bio Rad) was used according to the manufacturer’s instructions.

### Protein quantification

To determine the concentration of protein in vesicles, the Peterson assay was performed (21).

### Antibiotic susceptibility

Susceptibility of clinical strains to three different antibiotics (ceftazidime, meropenem, and amikacin) was determined by BD Phoenix at Le Scotte Hospital (Siena, Italy), while LS04 strain was sent for analysis to Centro Diagnostico Senese (Siena, Italy). The interpretation criteria recommended by EUCAST (Breakpoint Tables v. 14.0) were used for all strains.

### Dynamic light scattering

Vesicle samples were thawed, mixed with 1 vol of 1× PBS, and analyzed by dynamic light scattering (DLS) (Zetasizer Malvern). Conditions were 25°C; dispersant RI 1.33; dispersant viscosity 0.887, and dispersant dielectric constant 78.5.

### Transmission electron microscopy

For electron microscopy analysis, vesicle samples obtained with ExoBacteria OMV Isolation kit were washed with 1× PBS and concentrated (~5×) using an Amicon Ultra 0.5 centrifugal filter device (100 kDa cutoff). Later, 3 µL of each sample was loaded onto formvar-coated 400 mesh Cu grids (Ted Pella Inc., Redding, CA) for 2 min at RT. After removing the excess, 3 µL of 1% uranyl acetate (Polysciences Inc., Warrington, PA) in distilled water was added for 30 s. Finally, the samples were analyzed using a Thermo Fisher Scientific Tecnai G2 Spirit 120 kV transmission electron microscope (equipped with a TVIPS TemCam-F216 CMOS camera).

### UPGMA analysis and dendrogram generation

The phenotype data (pyoverdine production, colony size, pigment production, mucoid phenotype, ability to grow in M9 medium, resistance to ceftazidime, meropenem, and amikacin) was used to build a distance matrix. Jaccard coefficient and cluster analysis were done by UPGMA, using DendroUPGMA (http://genomes.urv.cat/UPGMA/) (22).

### Statistical analysis

GraphPad Prism 9 software was used to generate growth curves (biological triplicates) and DLS graphs.

## RESULTS

### Phenotypic characterization of clinical strains of *P. aeruginosa*

To analyze the phenotypic variability of clinical strains of *Pseudomonas aeruginosa*, we selected 13 clinical isolates and studied them alongside the reference strains *P. aeruginosa* PAO1 and ATCC 27853 (Table 1).

**Fig 1:**
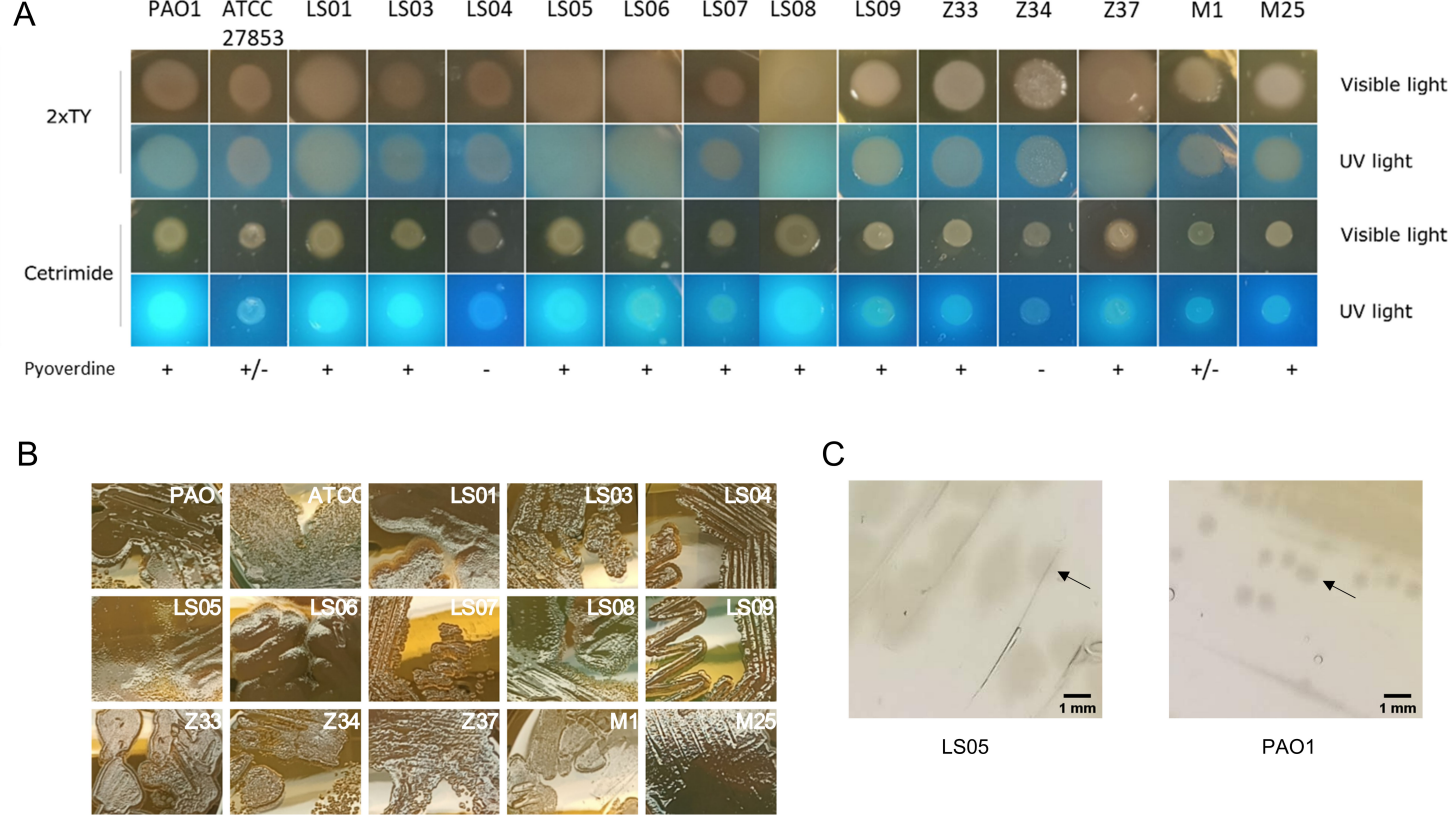
Growth phenotype of clinical strains. (**A**) Colony morphology assay. Growth and pyoverdine production were analyzed by growing *P. aeruginosa* strains in cetrimide and 2xTY agar plates. Pictures were taken under UV and visible light after incubation at 37°C for 24 h. (**B**) Growth phenotype in 2xTY agar. The strains were inoculated as streaks in agar plates and incubated at 37°C for 24 h. (**C**) Comparison of colony size between LS05 and PAO1 strains in cetrimide agar. Colonies are indicated with black arrows.

First, we performed a colony morphology assay. The strains were inoculated as droplets on 2xTY (a rich medium) and cetrimide agar [a selective medium for *Pseudomonas* which induces production of the fluorescent siderophore pyoverdine and other pigments such as pyocyanin (23)] and photographed under UV and visible light after overnight incubation. The results indicated that while all strains were able to grow on cetrimide agar, not all were able to produce the fluorescent siderophore pyoverdine (which is seen as white colonies without fluorescence) under these conditions (Fig. 1A). It was also possible to observe differences in growth behavior, i.e., after overnight incubation some strains grew poorly, while others produced large colonies, suggesting higher growth and/or different colony morphology. Indeed, when these strains were grown as streaks in 2xTY agar plates, several of these large colonies (LS01, LS05, LS06, LS08, and sometimes Z37) were observed to have undulating edges or borders that extended or spread beyond the edge of the colony (Fig. 1B and C).

To better focus on the production of pyoverdine and other pigments, the strains were grown as streaks on cetrimide, King A and King B agar plates, which are media commonly used to induce the production of different pigments in *Pseudomonas* species (15, 23), at 37°C for 48 h (to ensure proper growth of all strains). Our results indicated that most strains were able to produce pigments such as pyocyanin (blue pigment), pyoverdine (yellow-green fluorescent pigment), and pyorubin (red pigment, also known as “pyorubrin”) (Fig. S1A and B); however, strain LS04 from bronchial aspirate did not produce pyoverdine or any noticeable pigment under these test conditions. Also, most strains simultaneously produced pyoverdine and pyocyanin.

**Fig 2:**
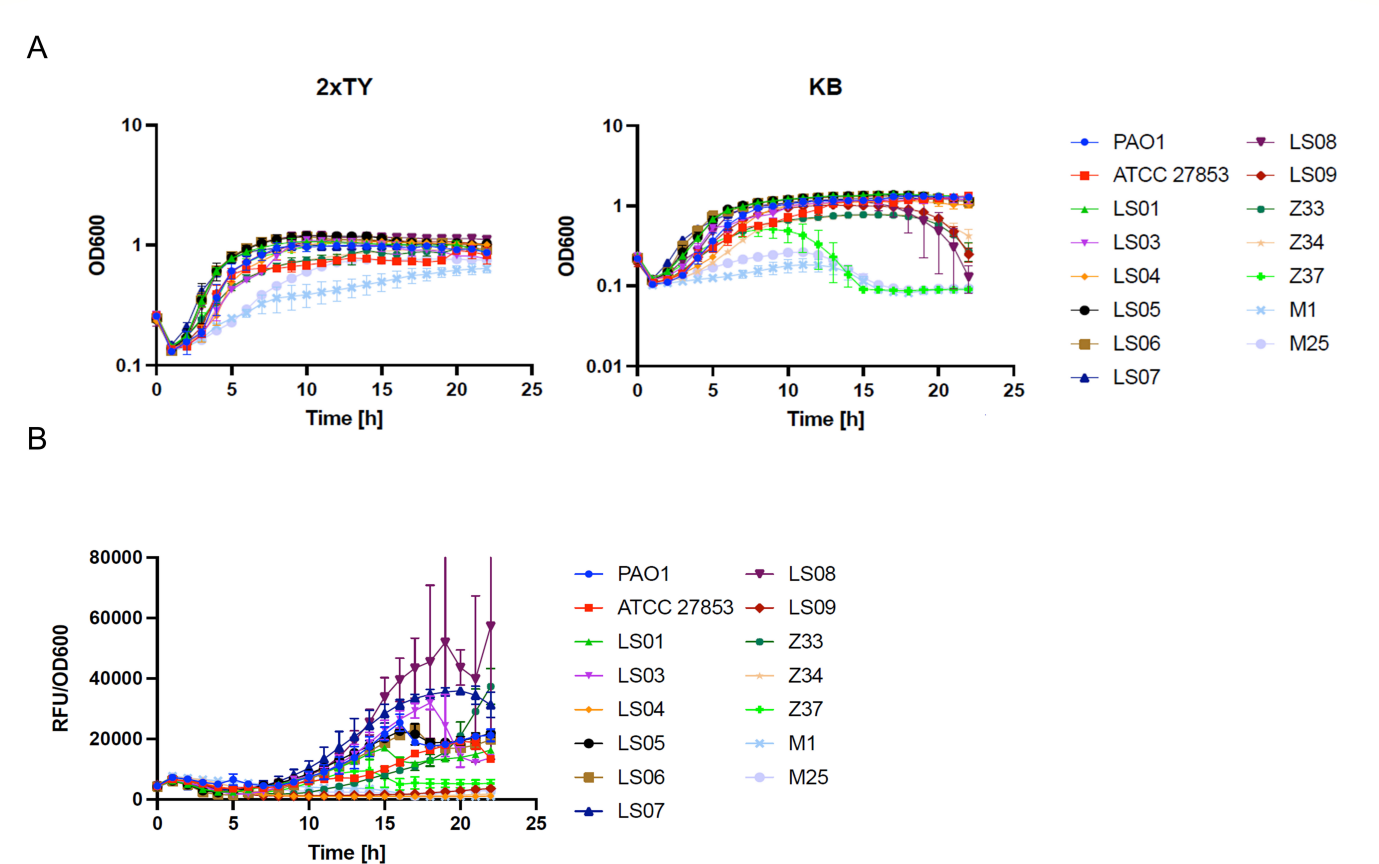
Growth phenotype of clinical strains of *P. aeruginosa*. (**A**) The growth pattern of clinical strains of *P. aeruginosa* in 2xTY and King B (KB) media was studied in 96-well plates incubated at 37°C for 22 h under continuous shaking. Pyoverdine production was analyzed by the measurement of fluorescence (excitation: 400 nm; emission: 455 nm). Graph (**B**) shows fluorescence normalized to growth (RFU/OD600). The experiment was performed in triplicate.

To further investigate pyoverdine production and to determine the growth phenotype of the strains, we performed growth curves in 2xTY and King B media for 22 h. OD600 and pyoverdine fluorescence (excitation: 400 nm; emission: 455 nm) were read every 30 min. Our results indicated that strains M1 and M25 grew slowly in 2xTY and barely grew in King B medium, while strain Z37 only showed slower growth in King B (Fig. 2A). The reduced growth rate of these strains is in line with previous reports on isolates from cystic fibrosis patients (24). On the other hand, strains LS09, LS08, Z33, and Z34 reached a maximum OD at about 13 h, showing a progressive reduction in absorbance from 18 h on. This was only seen in KB medium (Fig. 2A; Fig. S2). The difference in growth observed between these two culture conditions could be partly linked to pyoverdine production in some strains, as previously reported (25, 26). Indeed, the LS08 strain showed maximum pyoverdine production when its growth started to decline (Fig. 2A and B). However, pyoverdine production dynamics do not explain all cases, as seen for LS09 (a strain that barely produced pyoverdine in this assay) (Fig. 2B). Last of all, we corroborated the absence of pyoverdine production by strain LS04 grown under these conditions.

To continue with the phenotypic characterization, strains were inoculated into blood agar plates. Our results indicated that only three strains (Z37, M1, and M25) showed a mucoid phenotype, as previously reported for these isolates by Valzano et al. (19). In parallel, all strains showed a hemolytic phenotype after 48 h of incubation although only a few strains had produced hemolytic colonies after 24 h (Table S1).

Finally, we characterized the strains according to their susceptibility to three different antibiotics: ceftazidime, meropenem, and amikacin. We selected these antibiotics as representatives of three antibiotic classes (cephalosporins, carbapenems, and aminoglycosides, respectively) and because they are widely used in clinical settings. Our results indicated that some strains were multidrug-resistant, while most were sensitive to at least two antibiotics (Table 2).

**TABLE 2:** Antibiotic susceptibility of clinical strains of *Pseudomonas*^*b*^

Strain	Ceftazidime		Meropenem		Amikacin	
	MIC	Interpretation^*a*^	MIC	Interpretation	MIC	Interpretation
LS01	4	I	0.25	S	2	S
LS03	64	R	1	S	2	S
LS04	>32	R	8	I	>32	R
LS05	4	I	0.25	S	4	S
LS06	4	I	0.5	S	4	S
LS07	4	I	1	S	4	S
LS08	>16	R	>8	R	4	S
LS09	32	R	8	I	32	R
Z33	2	I	0.125	S	8	S
Z34	>256	R	16	R	16	S
Z37	64	R	2	S	8	S
M1	4	I	8	I	64	R
M25	2	I	<=0.06	S	4	S

^*a*^ Interpretation according to EUCAST.

^*b*^ R, resistant; I, susceptible, increased exposure; S, sensitive.

**Fig 3:**
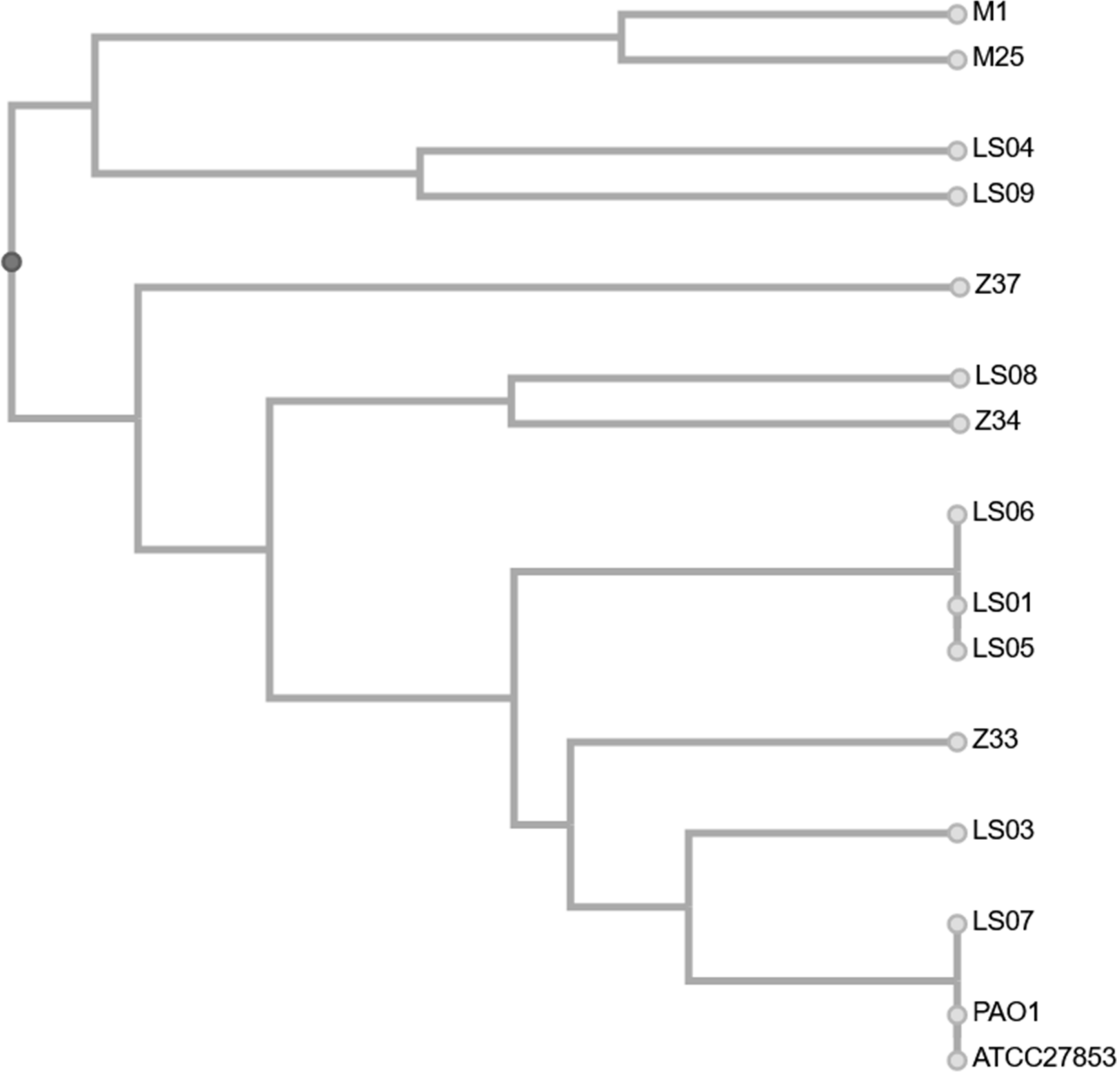
Dendrogram of phenotype characteristics of 15 *P*. *aeruginosa* strains. The dendrogram plot was generated using http://genomes.urv.cat/UPGMA/.

The phenotyping data were used to generate a distance matrix. The results (pyoverdine production, colony size, pigment production, mucoid phenotype, ability to grow in M9 medium, and resistance to ceftazidime, meropenem, and amikacin) were transformed into binary values and used for UPGMA analysis (Table S2). The resulting dendrogram indicated that only a few strains could be grouped according to phenotype, suggesting that our collection of samples was a heterogeneous group of isolates (Fig. 3). Taken together, these results indicate that our clinical strains were highly diverse isolates and illustrate the elevated phenotypic variability of clinical strains of *P. aeruginosa*.

### Selection of conditions for isolating vesicles

To isolate vesicles from *P. aeruginosa* strains at a quality level that allowed further molecular characterization by mass spectrometry and/or electron microscopy analysis, we performed the extraction from cultures of *P. aeruginosa* PAO1 reference strain, grown in King B medium (rich medium) and in M9 minimal medium supplemented with 0.4% glucose. For this particular step, solely to evaluate contamination of the sample with particles from the original culture medium and by way of comparison, we also used an ultrafiltration/ultracentrifugation protocol that was not developed for *P. aeruginosa* [and was, therefore, not optimized to exclude contaminants, such as bacterial flagella, for which there are other widely recommended protocols, e.g., the one described by Bauman and Kuehn (27)].

**Fig 4:**
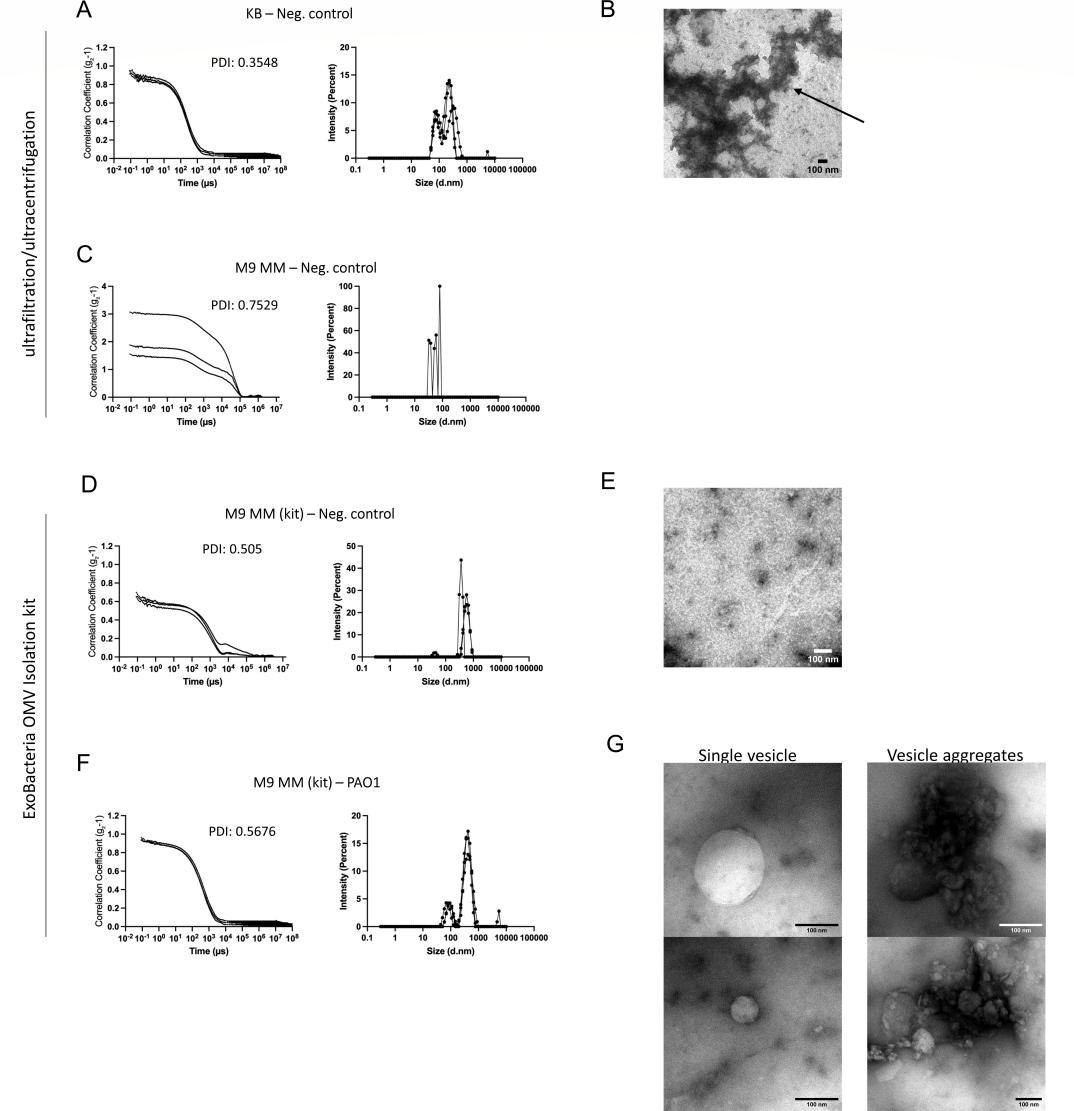
Analysis of growth media for vesicle purification by DLS showing (**A, C, D, F**) the results of sample analysis using DLS (Zetasizer). A correlogram (left) and a graph indicating size distribution by intensity (right) are shown for each set. (**A–C**) An ultracentrifugation/ultrafiltration protocol was used with (**A and B**) King B medium without bacteria (negative control for KB) and (**C**) M9 medium without bacteria (negative control for M9 medium). (**D–G**) ExoBacteria OMV Isolation Kit was used to purify vesicles/particles from M9 minimal medium: (**D–E**) without bacteria (negative control for M9 minimal medium) or (**F and G**) inoculated with *P. aeruginosa* PAO1 strain. The three curves in each graph represent the three readings of the instrument (triplicate). PDI, polydispersity index. (**B, E, G**) Electron microscopy images of (**B**) samples obtained with ultrafiltration/ultracentrifugation, protein aggregates (black arrow) of KB medium without bacteria; (**E**) samples obtained with ExoBacteria OMV Isolation kit M9 minimal medium (negative control); (**G**) vesicles of *P. aeruginosa* PAO1 grown in M9 minimal medium.

Our dynamic light scattering results showed a particle population peak for the negative control (KB medium without vesicles) using ultracentrifugation/ultrafiltration (Fig. 4A). The polydispersity index of this measurement, a dimensionless value indicating the level of sample heterogeneity (28), was 0.3548, suggesting a moderate particle size distribution. These particles are most likely protein aggregates generated by the peptone of the medium (which can also be seen after purification with ExoBacteria kit, Fig. 4B), as previously described for other media (29).

In contrast, the processing of M9 minimal medium without bacteria (negative control) with ultracentrifugation/ultrafiltration did not produce any noticeable peak by DLS analysis (triplicate readings by Zetasizer showed non-overlapping curves and a poor correlation function), implying that no specific particle population was detected (Fig. 4C). This suggested that M9 minimal medium was the best option for molecular analysis of vesicles, as we also did not observe any particle population in the negative control with the ExoBacteria kit (Fig. 4D and E). We, therefore, selected this condition for further purifications. Later, we used the ExoBacteria OMV isolation kit to purify vesicles of *Pseudomonas aeruginosa* PAO1 in M9 minimal medium. Our results indicated that the kit allowed purification of vesicles from just 25 to 30 mL of M9 minimal medium (Fig. 1F and G) although the protein concentration of the sample was low even after concentration (0.05 µg/µL).

### Vesicle isolation from clinical strains

**Fig 5:**
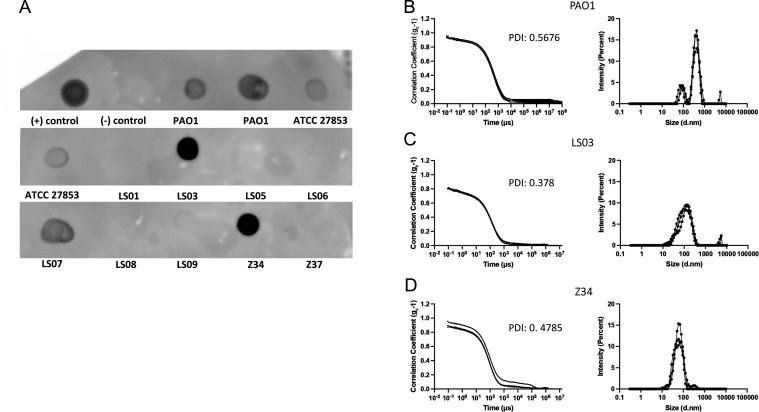
Immunodetection of porin OprF by dot blot and DLS analysis. (**A**) Dot blot using anti-OprF antibody. Samples eluted by Exobacteria OMV Isolation kit were spotted onto nitrocellulose membrane and used for dot blot. The positive (+) control corresponds to the first antibody solution and negative (−) control to blocking solution (3% BSA in TBS). Control strains were blotted twice (PAO1 from different purifications). (**B–D**) Analysis of eluates that showed the highest dot blot signals by DLS (Zetasizer). The curves obtained for (**B**) PAO1, (**C**) LS03, and (**D**) Z34 samples are shown. The three curves in each graph indicate the triplicate readings of the instrument. PDI, polydispersity index.

After characterizing enough diverse clinical strains and choosing the optimal culture conditions for vesicle purification, we used the rapid purification method to isolate vesicles. To do so, we grew all strains during the day on M9 minimal medium. After overnight incubation at 37°C, our first finding was that not all clinical strains were able to grow on M9 minimal medium (Table S3). In fact, four strains, including three isolates from patients with cystic fibrosis, failed to grow on this medium even after 72 h of incubation, suggesting either the presence of auxotrophies or an extremely slow-growing phenotype. We then purified vesicles from the 11 cultures by the rapid purification method. Screening for vesicles was first done by immunodetection of the porin OprF (a membrane protein of *P. aeruginosa*) using a dot blot. We used an antibody against this porin because although deletion mutants of *P. aeruginosa* have been described for OprF in biofilm-producing strains (30), its presence in clinical isolates seems to be quite constant due to its link to pathogenicity (31–33). The particle population profile of the samples was analyzed in parallel by DLS. Our results showed that only five strains, namely, ATCC 27853, LS03, LS07, Z34, and the control PAO1, were positive for OprF (indicating the presence of vesicle membranes in the eluates) (Fig. 5A). Further processing of the samples by dynamic light scattering revealed that only three (PAO1, LS03, and Z34) of these five eluates had a profile suggesting the presence of vesicles (Fig. 5B and D) although in the case of samples LS07 and ATCC 27853, the combination of vesicles with other molecules of different sizes could not be ruled out. Analysis of protein concentrations in positive samples ATCC 27853, LS03, LS07, and Z34 again showed very low concentrations: 0.08 µg/µL (after concentration), 0.156 µg/µL, 0.07 µg/µL, and 0.02 µg/µL, respectively. Finally, immunodetection of OprF in the positive clinical samples by western blot indicated that only two, LS03 and Z34, were positive (Fig. S3). This suggested that the dot blot result for LS07 was not specific (in line with the dynamic light scattering results).

## DISCUSSION

In this study, we analyzed 13 phenotypically diverse clinical strains of *P. aeruginosa*. The high level of phenotypic variability in clinical isolates of *P. aeruginosa* has been described in many other cases (14, 34–36) and may be a reason why purification of vesicles from these strains can be difficult. Indeed, some strains isolated from cystic fibrosis patients were particularly challenging due to their growth behavior. This is in line with previous reports that these isolates seem highly adapted to the host, as suggested by their typically slow growth (24) and auxotrophies (37).

Here, we showed that the culture medium selected to grow *P. aeruginosa* strains is essential to obtain vesicle samples of good quality for molecular biology. As we observed in our experiments, the KB culture medium is rich enough to ensure rapid bacterial growth but produces aggregates that can then be purified by ultrafiltration/ultracentrifugation and the rapid purification method. These aggregates can be seen by electron microscopy (Fig. 4B) and could be a serious problem for mass spectrometry if appropriate controls are not used. Indeed, other research groups, such as Le and colleagues (29), have described the importance of a culture medium that does not produce protein aggregates for the purification of bacterial vesicles. In this context, our results highlight the need to use different techniques, such as immunodetection, particle size analysis, and electron microscopy, to monitor for vesicles in the final samples since measuring protein or lipid concentrations cannot specifically distinguish vesicles from other carry-over products.

We found that the ExoBacteria OMV Isolation kit could only isolate vesicles from 4 of the 11 samples tested (including the two reference strains). The vesicle yield was low in all the positive eluates, suggesting that the kit does not seem to be convenient for clinical strains, where diverse phenotypes could influence its efficacy. This result may be due to specific phenotypes producing different quantities of OMVs of different quality although further analysis is needed to prove or disprove this speculation. Alternatively, this result could also be related to differences between the LPS composition of *P. aeruginosa* and *E. coli* (organism for which the ExoBacteria OMV isolation kit was originally designed). Our findings suggest that rapid vesicle purification methods do not currently seem to be the best option for clinical strains of *P. aeruginosa*, where other protocols (more laborious, but with better purity and yield) are recommended (27). Indeed, traditional protocols such as ultracentrifugation are helpful in getting rid of common contaminants of vesicle samples in *Pseudomonas*, such as flagella. In this context, the addition of a prior centrifugation step before using the kit in order to remove the flagella (16,000 × *g* for 30 min at 4°C) would be recommended (38, 39).

Taken together, our findings highlight the need to carefully standardize vesicle purification protocols in relation to the analyses to be performed downstream and the phenotypic and growth differences of the specific organisms to be grown. In conclusion, the present study contributes to our understanding of vesicle production and purification in *P. aeruginosa* strains and highlights the need for new rapid alternative/optimized protocols for phenotypically diverse populations.

## SUPPLEMENTAL MATERIAL

Supplemental material spectrum.00649-24-s0001.docx Tables S1 to S3; Fig. S1 to S3.

ASM does not own the copyrights to Supplemental Material that may be linked to, or accessed through, an article. The authors have granted ASM a non-exclusive, world-wide license to publish the Supplemental Material files. Please contact the corresponding author directly for reuse.
