# Supplementary material for: Analysis of the utility of a rapid vesicle isolation method for clinical strains of Pseudomonas aeruginosa
Source: Microbiol Spectr. 2024 Sep 9;12(10):e00649-24. doi: 10.1128/spectrum.00649-24 (PMC11448148; doi:10.1128/spectrum.00649-24)
Supplement: Supplemental material — Tables S1 to S3; Fig. S1 to S3. [file spectrum.00649-24-s0001.docx]

Supplementary material

Analysis of the utility of a rapid vesicle isolation method for clinical strains of *Pseudomonas aeruginosa*

Tania Henríquez^a^*, Francesco Santoro^a^, Donata Medaglini^a^, Lucia Pallecchi^a^, Ilaria Clemente^b^, Claudia Bonechi^b^, Agnese Magnani^b^, Eugenio Paccagnini^c^, Mariangela Gentile^c^, Pietro Lupetti^c^, Massimiliano Marvasi^d^, Alessandro Pini^a^, Luisa Bracci^a^, Chiara Falciani^a^.

^a^ Department of Medical Biotechnologies, University of Siena, Siena, Italy.

^b^ Department of Biotechnology, Chemistry and Pharmacy, University of Siena, Siena, Italy.

^c^ Department of Life sciences, University of Siena, Siena, Italy.

^d^ Department of Biology. University of Florence, Florence, Italy.

**Table S1.** Mucoid phenotype and hemolytic activity of clinical strains of *P. aeruginosa.*

| **Strain** | **Mucoid phenotype** | **Hemolysis - 24** | **Hemolysis- 48** |
| --- | --- | --- | --- |
| PAO1 | no | ++ | +++ |
| ATCC 27853 | no | + | +++ |
| LS01 | no | ++ | +++ |
| LS03 | no | + | +++ |
| LS04 | no | + | +++ |
| LS05 | no | + | +++ |
| LS06 | no | ++ | +++ |
| LS07 | no | + | +++ |
| LS08 | no | + | +++ |
| LS09 | no | + | +++ |
| Z33 | no | - | +++ |
| Z34 | no | - | +++ |
| Z37 | yes | + | +++ |
| M1 | yes | - | +++ |
| M25 | yes | - | +++ |

**Table S2. Results for phenotypic characterization transformed to binary data.**

| **Strains** | **Pyoverdine production in agar** | **Small colonies** | **Pigment production (color)** | **Mucoid phenotype** | **Growth in M9** | **Ceftazidime**  **resistance** | **Meropenem**  **resistance** | **Amikacin**  **resistance** |
| --- | --- | --- | --- | --- | --- | --- | --- | --- |
| PAO1 | 1 | 1 | 1 | 0 | 1 | 0 | 0 | 0 |
| ATCC | 1 | 1 | 1 | 0 | 1 | 0 | 0 | 0 |
| LS01 | 1 | 0 | 1 | 0 | 1 | 0 | 0 | 0 |
| LS03 | 1 | 1 | 1 | 0 | 1 | 1 | 0 | 0 |
| LS04 | 0 | 1 | 0 | 0 | 0 | 1 | 0 | 1 |
| LS05 | 1 | 0 | 1 | 0 | 1 | 0 | 0 | 0 |
| LS06 | 1 | 0 | 1 | 0 | 1 | 0 | 0 | 0 |
| LS07 | 1 | 1 | 1 | 0 | 1 | 0 | 0 | 0 |
| LS08 | 1 | 0 | 1 | 0 | 1 | 1 | 1 | 0 |
| LS09 | 0 | 1 | 1 | 0 | 1 | 1 | 0 | 1 |
| Z33 | 1 | 1 | 1 | 0 | 0 | 0 | 0 | 0 |
| Z34 | 0 | 1 | 1 | 0 | 1 | 1 | 1 | 0 |
| Z37 | 1 | 0 | 1 | 1 | 0 | 1 | 0 | 0 |
| M1 | 0 | 1 | 1 | 1 | 0 | 0 | 0 | 1 |
| M25 | 0 | 1 | 1 | 1 | 0 | 0 | 0 | 0 |

_1 = Positive result_

_0 = Negative result_

**Table S3.** Growth of clinical strains of *P. aeruginosa* in M9 minimal medium supplemented with glucose after overnight incubation (before vesicle purification with ExoBacteria Kit).

| **Strain** | **OD600** |
| --- | --- |
| PAO1 | 0.7 |
| ATCC 27853 | 0.417 |
| LS01 | 0.520 |
| LS03 | 0.875 |
| LS04 | No growth |
| LS05 | 1.620 |
| LS06 | 0.868 |
| LS07 | 0.89 |
| LS08 | 0.958 |
| LS09 | 0.792 |
| Z33 | No growth |
| Z34 | 1.118 |
| Z37 | 0.923 |
| M1 | No growth |
| M25 | No growth |


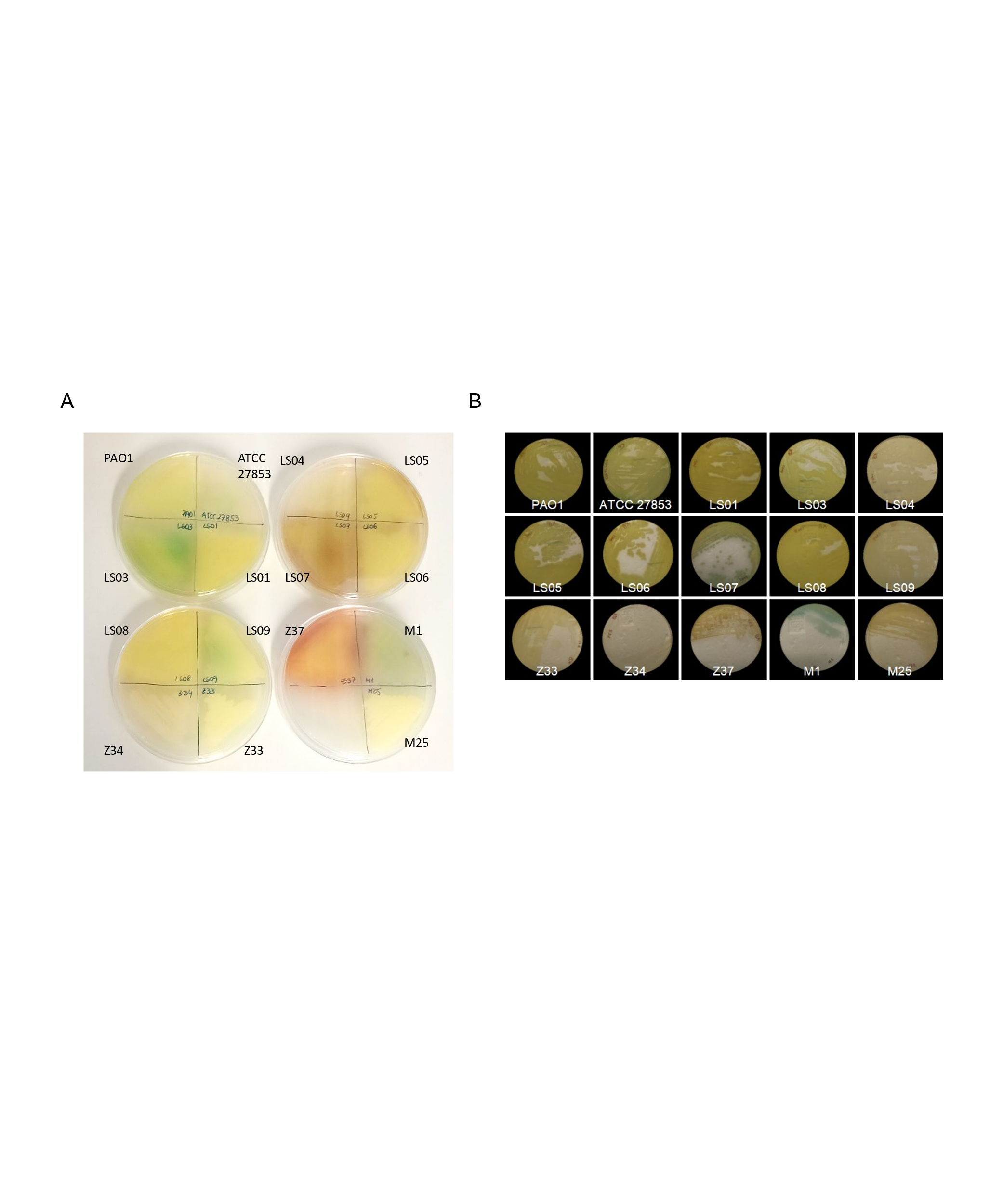


**Fig. S1.** Analysis of the production of pigments in clinical strains. Strains of *P. aeruginosa* were grown in (A) cetrimide and (B) King B agar plates and incubated at 37°C for 48 h. In the case of (B), a montage was generated with ImageJ.


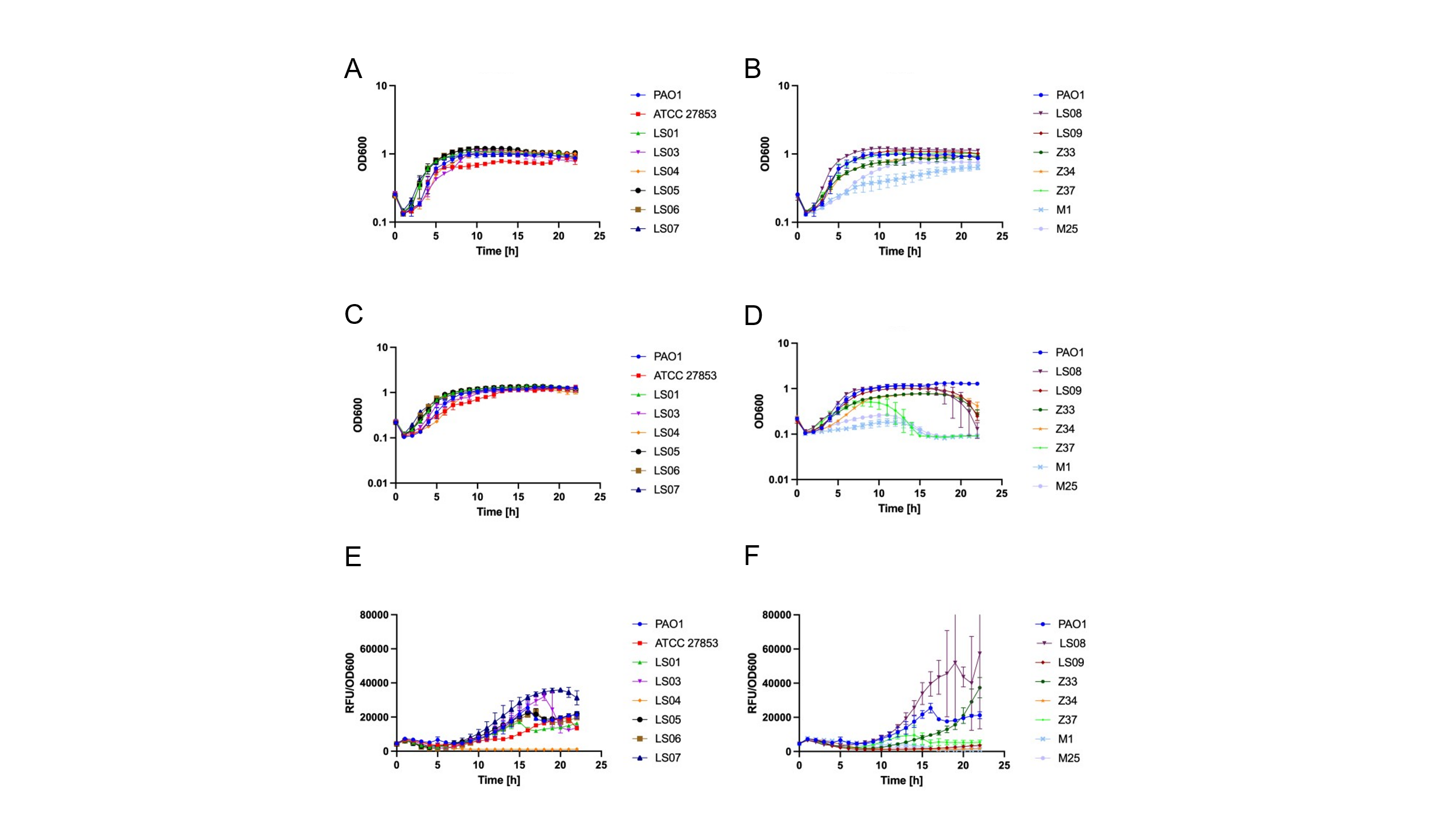


**Fig. S2.** Growth and fluorescence of *P. aeruginosa* strains. (A-B) Growth in 2xTY medium. (C-D) Growth in KB medium. (E-F) Measurement of the fluorescence of the strains in KB medium normalized by growth (OD600). Growth behavior of clinical strains of *P. aeruginosa* in 2xTY and King B (KB) media was studied in 96-well plates incubated at 37°C for 22 h with continuous shaking. Pyoverdine production was analyzed through the measurement of fluorescence (excitation: 400 nm; emission: 455 nm). The experiment was performed in triplicate.


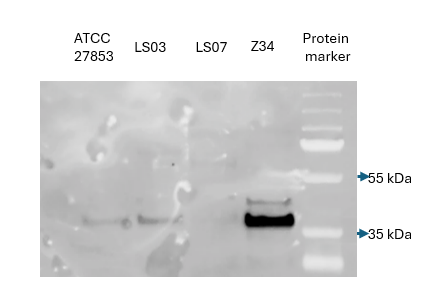


**Fig. S3**. Immunodetection of OprF porin in vesicle samples of clinical strains LS03, LS07 and Z34. Eluates from ExoBacteria OMV isolation Kit were used for SDS-PAGE and Western blot analysis using an antibody against *P. aeruginosa* OprF*.*
